# Bibliometric Analysis of Patient‐Controlled Intravenous Analgesia Research From 2014 to April 2026: Global Trends, Hotspot Evolution, and Future Directions

**DOI:** 10.1155/prm/2497105

**Published:** 2026-09-15

**Authors:** Zheng-Yuan Duan, Shuai-Yu Zhu, Qi-Jun Li, Xin-Ya Cao, Xing-Cheng Wang, Qiu-Xia Xiao, Liu-Lin Xiong

**Affiliations:** ^1^ Department of Anesthesiology, The Third Affiliated Hospital of Zunyi Medical University (The First People’s Hospital of Zunyi), Zunyi 563000, Guizhou, China; ^2^ The School of Professional Education and Executive Development, The Hong Kong Polytechnic University, Hong Kong 999077, China, polyu.edu.hk

**Keywords:** bibliometric analysis, multimodal analgesia, opioid-sparing strategy, patient-controlled intravenous analgesia, postoperative pain

## Abstract

**Background:**

Patient‐controlled intravenous analgesia (PCIA) remains a cornerstone in managing moderate to severe postoperative pain. The shift toward multimodal analgesia, opioid‐sparing protocols, and enhanced recovery after surgery has steadily broadened the scope of PCIA‐related investigation. Despite this growth, a clear synthesis of the global research landscape, underlying knowledge structure, and the trajectory of emerging themes is still lacking.

**Methods:**

The Web of Science Core Collection was searched for English‐language original articles and reviews on PCIA published between January 1, 2014, and April 6, 2026. Bibliometric and visualization analyses were performed using Microsoft Excel, VOSviewer, CiteSpace, Bibliometrix, and SCImago Graphica to examine publication trends, contributing countries/regions, institutions, authors, journals, keywords, and cocited references.

**Results:**

The full dataset retrieved through April 6, 2026, included 492 publications involving 33 countries/regions, 607 institutions, and 2675 authors. In the analysis of complete publication years from 2014 to 2025, PCIA‐related research showed a fluctuating but overall upward trend. China, South Korea, and the United States emerged as the leading contributors. Although China produced the largest volume of publications, the United States held a more central position within the international collaboration network. The most prolific journals were Medicine, BMC Anesthesiology, and Journal of Pain Research. Research hotspots have progressively moved beyond opioid analgesia and efficacy evaluation to multimodal analgesia, opioid‐sparing strategies, safety optimization, integration with regional blocks, procedure‐specific analgesia, and patient‐centered outcomes.

**Conclusion:**

This study delineates the global scientific landscape and the evolution of research hotspots in PCIA, providing bibliometric evidence to inform future study design, strengthen cross‐regional collaboration, and shape individualized perioperative analgesic strategies.

## 1. Introduction

Postoperative pain continues to be one of the most prevalent and difficult clinical challenges in perioperative care. Inadequately controlled postoperative pain not only reduces patient comfort and satisfaction, but may also delay early mobilization, slow functional recovery, prolong hospital stays, increase healthcare resource consumption, and contribute to long‐term adverse outcomes such as persistent postoperative pain [1, 2]. Even as perioperative analgesic strategies have advanced, finding an acceptable balance among effective pain relief, reduced opioid exposure, fewer adverse events, and improved patient experience remains a central tension in postoperative pain management. Patient‐controlled intravenous analgesia (PCIA) is a widely used technique for managing moderate to severe postoperative pain. Unlike conventional as‐needed dosing, PCIA allows patients to self‐administer additional analgesics in response to their own pain intensity, within preset safety parameters, thereby improving the timeliness and personalization of drug delivery [3, 4].

With the widespread implementation of multimodal analgesia and enhanced recovery after surgery, the research focus of PCIA has moved well beyond opioid efficacy alone. Investigations now span combination therapies with nonopioid adjuvants, opioid‐sparing regimens, adverse effect management, procedure‐specific analgesia, integration with regional blocks, and patient‐reported outcomes [5–7]. This shift signals that PCIA has evolved from a stand‐alone drug‐delivery technique into a meaningful component of comprehensive perioperative analgesic care. Yet the rapid growth of PCIA‐related research has also introduced new challenges. The literature has largely focused on specific drug combinations, dosing schedules, surgical populations, or individual clinical outcomes, leaving the evidence base somewhat fragmented. Previous systematic reviews and meta‐analyses have appraised the efficacy and safety of certain PCIA regimens, but they have typically addressed narrow interventions or tightly framed clinical questions. Consequently, they offer limited capacity to reveal, from a macroscopic viewpoint, the overarching knowledge structure, major contributors, international collaboration patterns, evolution of research hotspots, and future trends in this field. Accordingly, a systematic visual‐analytic approach is needed to map the research trajectory of PCIA.

Bibliometric analysis integrates mathematical, statistical, and bibliographic methods to quantitatively assess publication output, academic impact, collaboration networks, cocitation structures, and keyword evolution trends within a specific field [8, 9]. Compared with traditional narrative reviews, bibliometric analysis can provide a more objective and reproducible evidence map drawn from large‐scale literature data at the levels of knowledge structure and research trends.

Given the sustained importance of PCIA in perioperative pain management and the continuous broadening of its research themes, this study conducted a bibliometric and visualization analysis of global PCIA‐related literature published from January 1, 2014 to April 6, 2026, using the Web of Science Core Collection (WOSCC) database. The objectives were to identify the major contributing countries/regions, institutions, authors, and journals in this field; map collaboration and cocitation networks; and to uncover research hotspots, knowledge gaps, and the evolution of frontier topics, thereby providing a reference for future clinical study design, academic collaboration, and optimization of perioperative analgesic strategies.

## 2. Methods

### 2.1. Data Sources and Search Strategy

**FIGURE 1:**
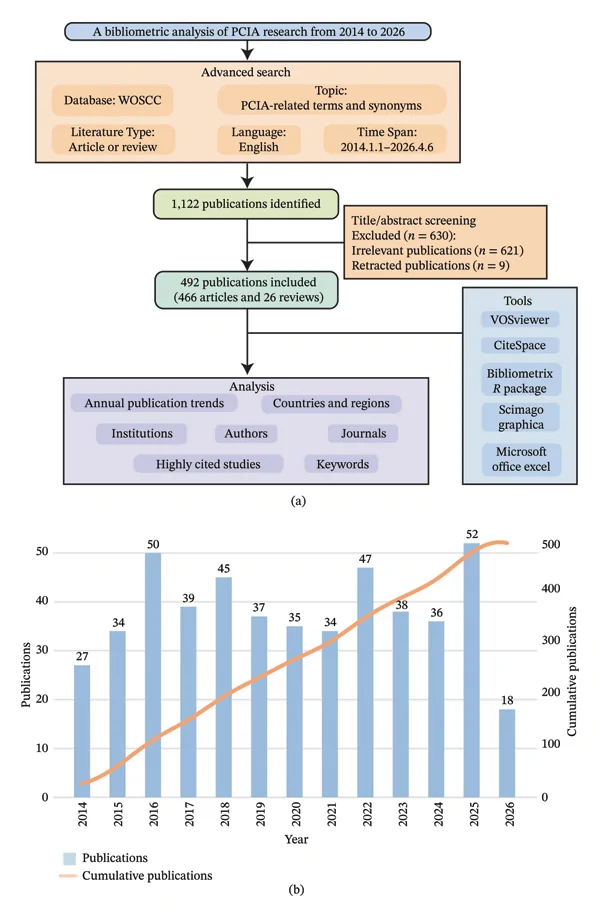
Literature screening workflow and publication trends of PCIA research from 2014 to 2026. (a) Flowchart of literature retrieval, screening, and final inclusion. (b) Annual publication output and cumulative publication growth in PCIA research during the study period.

The bibliographic data were retrieved from the WOSCC database. We chose WOSCC as the sole data source because it is a standard platform in bibliometric research, offering consistently formatted full records and cited reference data. Previous high‐impact bibliometric studies have likewise built their analyses of countries/regions, institutions, authors, keywords, and cocitation networks on WOSCC records processed with VOSviewer and CiteSpace [10, 11]. The database also provides standardized citation details, author affiliations, country/region information, and export formats that are directly compatible with these tools—all of which are indispensable for citation‐based bibliometric analysis. The search strategy relied on the topic search (TS) field, using the following search query: TS = (“patient controlled intravenous analgesia” OR “patient‐controlled intravenous analgesia” OR “intravenous patient‐controlled analgesia” OR “intravenous PCA” OR “IV PCA” OR “IV‐PCA” OR “IV patient‐controlled analgesia” OR “opioid PCA” OR “patient‐controlled intravenous opioid analgesia” OR “PCIA” OR “PCIA pump”) [12–15]. The search was limited to English‐language original articles and reviews published between January 1, 2014, and April 6, 2026. The literature screening process is illustrated in Figure 1a. All records meeting the inclusion criteria were downloaded in plain text format for subsequent bibliometric and visualization analyses.

### 2.2. Literature Screening

Two authors (Duan and Zhu) independently extracted core details from each record, including title, abstract, keywords, author names, affiliations, countries/regions, references, journal name, and publication year. Disagreements were resolved through consultation and discussion to ensure alignment with the inclusion criteria. The initial search retrieved 1122 records, restricted to original articles and reviews. After screening titles and abstracts, 621 off‐topic studies were excluded, and a further 9 retracted publications were removed, leaving 492 eligible publications for analysis. All search and screening procedures were completed on April 8, 2026, to reduce the risk of bias from dynamic database updates. Because the study drew exclusively on publicly available data, institutional review board approval was not necessary.

### 2.3. Data Analysis and Visualization

Using the final set of included publications, we mapped the trajectory of PCIA‐related research output. Data were organized and curated in Microsoft Excel 2024, which was also used to tally the number of publications per country or region and to compute the compound annual growth rate (CAGR) of yearly publication counts from 2014 to 2025 [16]. As the literature search was conducted in April 2026, that year did not represent a complete publication window and was therefore not included in the CAGR calculation. The CAGR was determined as follows:

(1) CAGR=VnV01/n−1×100%.

Here, *V*_0_ represents the publication count in the initial year, *V*_*n*_ represents the publication count in the final year, and *n* represents the time interval between the initial and final years. In our data, *V*_0_ was 27 article publications in 2014, *V*_*n*_ was 52 publications in 2025, and *n* = 11. The geographic distribution of national output was visualized with VOSviewer 1.6.20 (https://www.vosviewer.com/download) combined with SCImago Graphica 1.0.46 (https://www.graphica.app). We also used CiteSpace 6.3.R1 (https://CiteSpace.podia.com/) and the *R* package “bibliometrix” (version 5.2.1, https://www.bibliometrix.org) to analyze and visualize institutions, authors, journal dual‐map overlays, references, and keywords across the PCIA literature. For CiteSpace, the time slice was set to 1 year; the minimum burst strength threshold was set to 0.5 for keyword burst analysis and 1.0 for reference burst analysis; and the clustering parameter K for reference cocitation clustering was set to 13. Impact factor data were taken from the 2025 edition of Journal Citation Reports. All analytical parameters, thresholds, and visualization settings were standardized and recorded to support reproducibility.

## 3. Results

### 3.1. Annual and Cumulative Publication Output

From 2014 to 2025, the annual publication output in the PCIA field followed a fluctuating yet generally upward trajectory. The yearly output rose from 27 publications in 2014 to 52 publications in 2025. Despite year‐to‐year variability, scholarly activity remained fairly sustained, with comparatively productive years in 2016 (50 publications), 2018 (45 publications), 2022 (47 publications), and 2025 (52 publications). The cumulative number of publications reached 492 by the retrieval date (Figure 1b). To quantify this trend, we calculated the CAGR for the complete publication years from 2014 to 2025, which was approximately 6.14%, underscoring a consistently active research output over the past decade.

### 3.2. Country/Region Analysis and International Collaboration

**FIGURE 2:**
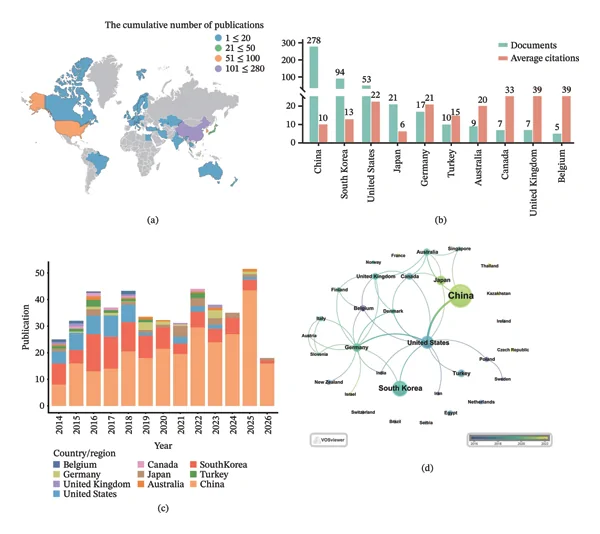
Country/region‐level bibliometric characteristics of PCIA research from 2014 to 2026. (a) Global geographic distribution of cumulative publications by countries/regions. (b) Top 10 countries/regions ranked by number of publications and average citations per document. (c) Annual publication output of the top 10 most productive countries/regions. (d) Country/region coauthorship network generated by VOSviewer. Node size reflects publication volume, node color indicates the average publication year, and link thickness represents collaboration strength.

A total of 33 countries/regions contributed to PCIA‐related research, with the majority concentrated in Asia, North America, and Europe (Figure 2a). China produced the most publications (278 publications), accounting for a large share of the total, followed by South Korea (94 publications) and the United States (53 publications). Japan, Germany, Turkey, Australia, Canada, the United Kingdom, and Belgium also ranked among the major contributors (Figure 2b). In terms of academic impact, publication output did not fully map onto citation influence: China averaged 10 citations per paper, whereas Belgium and the United Kingdom each averaged 39, Canada 33, the United States 22, and Germany 21 (Figure 2b). Annual publication trends showed that China remained the dominant source of publications across most years, while the publication outputs from South Korea and the United States stayed relatively steady (Figure 2c). The international collaboration network revealed that the United States held a central position, with the highest total link strength. Germany also showed relatively broad collaborative links. Although China was the most productive country, its collaborative links were relatively concentrated, anchored by a strong bilateral partnership with the United States—the tightest collaborative tie in the network. China also maintained connections with Japan and Singapore. South Korea, despite ranking second in publication output, had limited international links, largely restricted to collaborations with the United States and Germany (Figure 2d).

### 3.3. Institutional Publication Output and Collaboration Analysis

**FIGURE 3:**
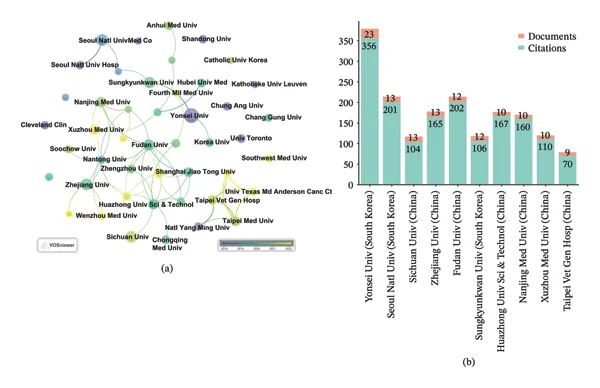
Institution‐level bibliometric characteristics of PCIA research from 2014 to 2026. (a) Institutional coauthorship network generated by VOSviewer. Node size reflects publication volume, node color indicates the average publication year, and links represent collaborative relationships between institutions. (b) Top 10 most productive institutions ranked by number of publications and total citations.

In total, 607 institutions contributed to PCIA‐related studies, 46 of which published at least 4 papers. East Asia emerged as the dominant hub of PCIA research. Within the institutional collaboration network, several centers stood out for their active collaborative ties, including Taipei Veterans General Hospital, Taipei Medical University, The University of Texas MD Anderson Cancer Center, National Yang Ming University, and Fudan University. The temporal overlay further indicated that recent activity has increasingly focused on exchanges among institutions within Asia (Figure 3a). Among the top 10 most productive institutions, Yonsei University and Seoul National University College of Medicine demonstrated particularly strong citation performance. Yonsei University ranked first with 23 publications and 356 citations, followed by Seoul National University College of Medicine (13 publications and 201 citations). The remaining top‐tier institutions were predominantly from China, such as Sichuan University (13 publications and 104 citations), Zhejiang University (13 publications and 165 citations), and Fudan University (12 publications and 202 citations) (Figure 3b).

### 3.4. Author Publication Output, Influence, and Collaboration Analysis

**FIGURE 4:**
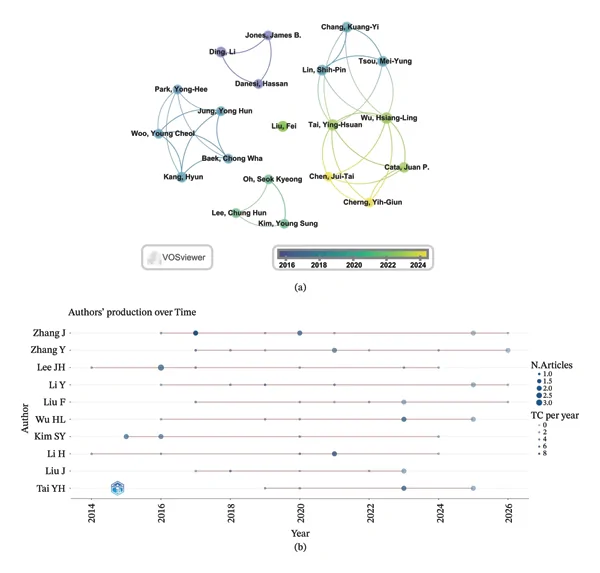
Author‐level bibliometric analysis of PCIA research from 2014 to 2026. (a) Coauthorship network of authors visualized with VOSviewer. Node size is proportional to the number of publications; node color represents the average publication year. Connecting lines indicate collaborative links between authors. (b) Timeline of authors’ production over time. Bubble size represents the number of articles published by each author; bubble color indicates total citations per year.

Of the 2675 authors contributing to PCIA research, 25 authors published at least 4 papers. Tai, Ying‐Hsuan and Wu, Hsiang‐Ling were the most productive authors, each contributing 6 publications; the remaining 9 highly productive authors each published 5 papers. Among those with five publications, Kang, Hyun recorded the highest citation count (86 citations), followed by Cata, Juan P. (83 citations) and Duan, Guang‐You (50 citations), underscoring that scholarly impact differed meaningfully even within this productive group (Table 1). In the collaboration network, Tai, Ying‐Hsuan and Wu, Hsiang‐Ling were positioned at the center, combining high output with dense collaborative ties. The cluster involving Cata, Juan P. has been particularly active in recent years, while Kang, Hyun emerged as a highly influential author within a separate core group (Figure 4a). The temporal distribution of author activity further indicated that Zhang J, Zhang Y, and Lee JH had the longest research engagement, whereas Tai, Ying‐Hsuan, Wu, Hsiang‐Ling, and Liu, Fei have become more prominent in the recent literature (Figure 4b).

**TABLE 1:** Authors with at least five publications in PCIA research.

Rank	Author	Documents	Citations	Average citations
1	Tai, Ying‐Hsuan	6	43	7.2
2	Wu, Hsiang‐Ling	6	43	7.2
3	Kang, Hyun	5	86	17.2
4	Cata, Juan P.	5	83	16.6
5	Wang, Wei	5	59	11.8
6	Duan, Guang‐You	5	50	10.0
7	Chang, Kuang‐Yi	5	46	9.2
8	Lin, Shih‐Pin	5	46	9.2
9	Tsou, Mei‐Yung	5	46	9.2
10	Liu, Fei	5	35	7.0
11	Ding, Li	5	25	5.0

### 3.5. Journal Publication Output, Influence, and Citation Network Analysis

**FIGURE 5:**
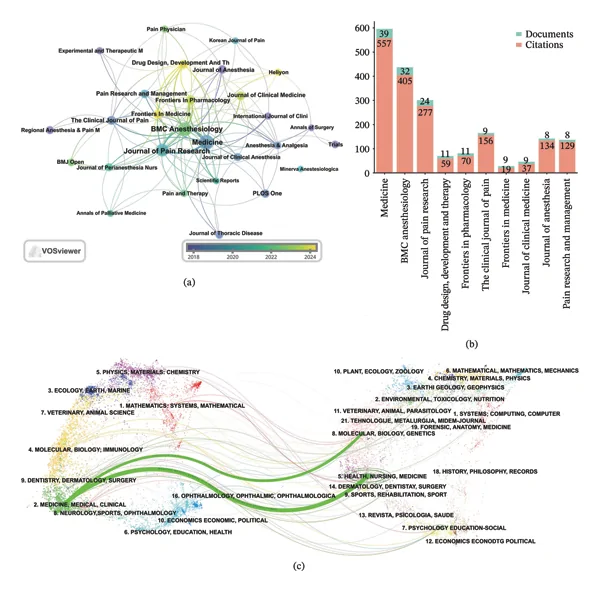
Journal‐level bibliometric characteristics of PCIA research from 2014 to 2026. (a) Journal citation network generated by VOSviewer. Node size reflects publication output, and links represent citation relationships between journals. (b) Top 10 journals ranked by number of publications and total citations. (c) Dual‐map overlay of citing journals (left) and cited journals (right), showing the major cross‐disciplinary citation pathways in PCIA research.

A total of 189 journals published PCIA‐related studies, among which 28 journals contributed at least 4 publications. Within the journal citation network, BMC Anesthesiology, Medicine, and Journal of Pain Research occupied central positions and maintained strong citation links with outlets such as Frontiers in Pharmacology, Journal of Anesthesia, and Pain Research and Management (Figure 5a). Among the top 10 most prolific journals, Medicine led in both publication count (39 publications) and total citations (557 citations), followed by BMC Anesthesiology (32 publications, 405 citations) and Journal of Pain Research (24 publications, 277 citations) (Figure 5b). Of these top 10 journals, 4 were ranked in Q1 and 6 in Q2. Clinical Journal of Pain, Journal of Anesthesia, and Pain Research and Management recorded particularly high citation averages per paper (Table 2). The dual‐map overlay showed that the citing journals in PCIA research were mainly distributed in the fields of Medicine, Medical, and Clinical, whereas the cited journals were primarily concentrated along disciplinary paths such as Health, Nursing, Medicine and Molecular, Biology, Genetics (Figure 5c).

**TABLE 2:** Top 10 journals by publication volume in the field of PCIA.

Journal	Publications	Average citations	IF (2025	JCR (2025)
Medicine	39	14.28	2	Q2
BMC Anesthesiology	32	12.66	3.2	Q2
Journal of Pain Research	24	11.54	3.1	Q2
Drug Design, Development and Therapy	11	5.36	6.1	Q1
Frontiers in Pharmacology	11	6.36	5.4	Q1
Clinical Journal of Pain	9	17.33	3.2	Q2
Frontiers in Medicine	9	2.11	3.6	Q1
Journal of Clinical Medicine	9	4.11	3.3	Q1
Journal of Anesthesia	8	16.75	2.8	Q2
Pain Research and Management	8	16.13	3.1	Q2

### 3.6. Keyword Co‐Occurrence, Evolution, and Research Hotspot Analysis

**FIGURE 6:**
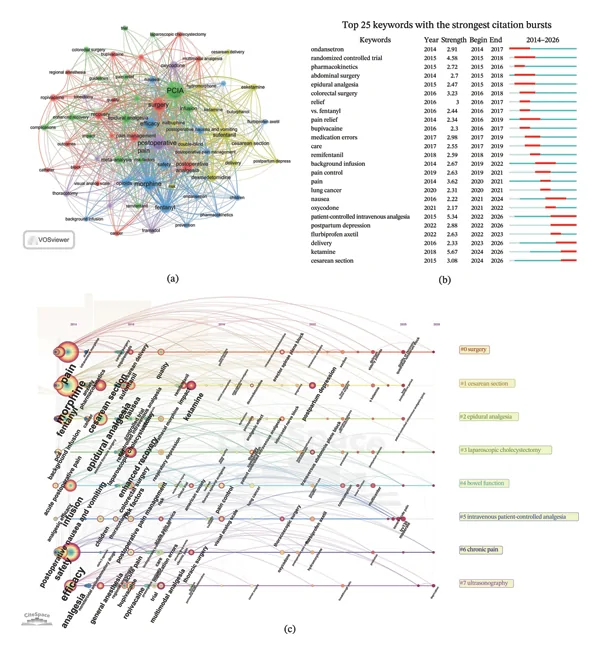
Keyword‐level bibliometric analysis of PCIA research from 2014 to 2026. (a) Co‐occurrence network of keywords visualized with VOSviewer. Node size is proportional to the frequency of occurrence; node color represents different keyword clusters. Connecting lines indicate co‐occurrence links between keywords. (b) Top 25 keywords with the strongest bursts. “Year” indicates the year in which the keyword first appeared in the dataset. Begin and end represent the burst time interval. The diagram on the right: The blue line indicates the overall time interval, and the red segment indicates the burst period. (c) Timeline view of keyword evolution and clusters from 2014 to 2026 visualized with CiteSpace. Nodes represent keywords; lines show co‐occurrence relationships over time. The right panel lists the major keyword clusters with representative terms.

The co‐occurrence analysis identified a core of high‐frequency keywords revolving around PCIA (217 occurrences), postoperative pain (142 occurrences), morphine (129 occurrences), surgery (120 occurrences), efficacy (91 occurrences), and fentanyl (77 occurrences) (Table 3). Other terms such as sufentanil, postoperative analgesia, epidural analgesia, opioids, and dexmedetomidine also displayed considerable frequency and strong co‐occurrence links (Figure 6a). Burst detection revealed distinct temporal phases in research emphasis. Earlier bursts were dominated by randomized controlled trial, abdominal surgery, epidural analgesia, pharmacokinetics, bupivacaine, and pain relief. This was followed by a wave of bursts that included background infusion, pain control, pain, nausea, and oxycodone. In recent years, burst activity has shifted toward PCIA, postpartum depression, delivery, ketamine, cesarean section, and flurbiprofen axetil; among these, ketamine and PCIA stood out with particularly high burst intensities (Figure 6b). CiteSpace clustering and timeline analysis identified 8 thematic keyword clusters: #0 surgery, #1 cesarean section, #2 epidural analgesia, #3 laparoscopic cholecystectomy, #4 bowel function, #5 intravenous patient‐controlled analgesia, #6 chronic pain, and #7 ultrasonography. The timeline view further suggested that cesarean section, ketamine, esketamine, hydromorphone, postpartum depression, and enhanced recovery are among the terms that have surfaced more recently, pointing to evolving research priorities (Figure 6c).

**TABLE 3:** Top 10 high‐frequency keywords in PCIA research identified by VOSviewer.

Rank	Keyword	Occurrences
1	PCIA	217
2	Postoperative pain	142
3	Morphine	129
4	Surgery	120
5	Efficacy	91
6	Fentanyl	77
7	Sufentanil	63
8	Postoperative analgesia	60
9	Epidural analgesia	49
10	Opioids	49

### 3.7. Reference Analysis

**FIGURE 7:**
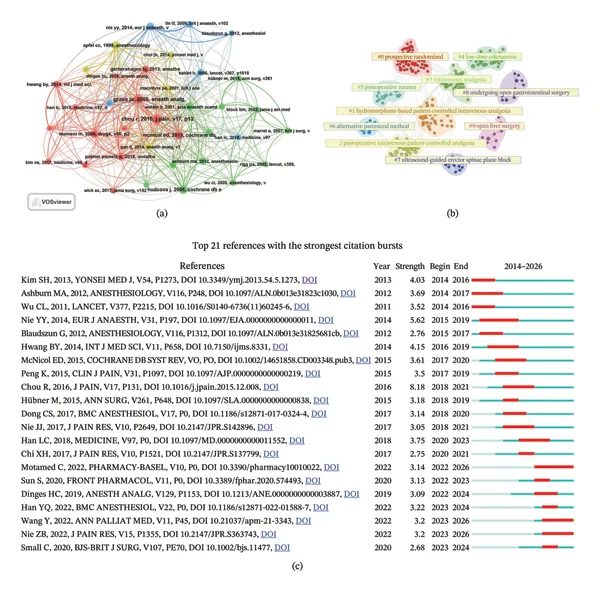
Reference‐level bibliometric characteristics of PCIA research from 2014 to 2026. (a) Reference cocitation network generated by VOSviewer. Node size reflects citation frequency, node color indicates cluster membership, and links represent cocitation relationships between references. (b) Cluster visualization of highly cocited references generated by CiteSpace, showing the major thematic clusters with representative labels. (c) Top 21 references with citation bursts.

A total of 10,396 cited references were identified across the included publications, among which 37 were cocited 12 or more times. The references with the highest cocitation frequencies included Chou R, 2016; Grass JA, 2005; McNicol ED, 2015; Apfel CC, 1999; and Momeni M, 2006, which were represented by larger nodes in the network. In the cocitation network, these highly cocited references occupied central positions and formed dense connections with multiple related references (Figure 7a). These highly cocited references centered on topics such as postoperative pain management guidelines, the principles and efficacy of PCIA, risk assessment of postoperative nausea and vomiting (PONV), and comparisons of different analgesic strategies. CiteSpace clustering analysis identified 10 major themes, including #0 prospective randomized, #1 hydromorphone‐based PCIA, #2 postoperative intravenous patient‐controlled analgesia, #3 intravenous analgesia, #4 low‐dose esketamine, #5 postoperative nausea, #6 alternative parenteral method, #7 ultrasound‐guided erector spinae plane block, #8 undergoing open gastrointestinal surgery, and #9 open liver surgery (Figure 7b). Reference burst analysis identified several references with strong temporal surges in citations. Between 2014 and 2019, prominent burst references included Kim SH, 2013; Ashburn MA, 2012; Wu CL, 2011; Nie YY, 2014; and Hwang BY, 2014. References with bursts persisting through 2026 included Motamed C, 2022, Wang Y, 2022, and Nie ZB, 2022 (Figure 7c).

## 4. Discussion

### 4.1. Global Knowledge Structure and Development Trajectory of PCIA Research

Our analysis revealed a fluctuating yet generally upward trend in PCIA‐related research from 2014 to 2025. The CAGR, calculated for the complete years 2014–2025, reached 6.14%, underscoring sustained scholarly interest in PCIA as a perioperative pain management topic. Globally, research output was clearly concentrated in a few countries, yet academic influence varied considerably across them. China led in publication volume, likely driven by its large surgical caseload, widespread adoption of PCIA for postoperative analgesia, and growing demand for perioperative pain management research. Strikingly, publication volume, citation impact, and collaborative centrality did not always align. Despite its dominant output, China’s average citations per paper and its position in international collaboration networks did not match its productivity. South Korea produced considerably fewer papers than China, yet several of its institutions and authors figured prominently in collaboration networks and garnered high citation counts, indicating a notable representativeness of its PCIA‐related clinical research. The United States, though ranking third in output, held a more central node in the global collaboration network, reflecting strong cross‐regional connectivity. Future work should therefore prioritize multicenter, cross‐regional collaborations with standardized protocols, thereby enhancing research quality, evidential strength, and the generalizability of results.

Turkey, ranking sixth worldwide in publication volume, demonstrated notable activity in PCIA and perioperative analgesia research. Yet, it remained peripheral in the international collaboration network, with its contributions largely reflected in independent or regional publications. This pattern underscores that PCIA, being a mature modality adaptable to diverse healthcare resource settings, garners sustained research interest not only in high‐income countries but also in middle‐income and regional health systems.

Journal distribution patterns, the dual‐map overlay, and the keyword and cocitation analyses collectively pointed to a distinctly interdisciplinary character. PCIA research now spans anesthesiology and pain medicine, pharmacology, nursing management, perioperative quality improvement, and patient‐centered outcomes such as experience and recovery quality. Keyword evolution and burst detection reinforced this shift. Early work revolved around opioids, analgesic efficacy, dosing regimens, and background infusions. More recent themes—dexmedetomidine, esketamine, PONV, cesarean section, regional blocks, and enhanced recovery—have gained considerable momentum. This signals a broadening of the PCIA research agenda from opioid‐centric efficacy comparisons toward a more holistic paradigm that integrates analgesic effectiveness, safety, recovery quality, and patient experience. Such a trajectory aligns with recent bibliometric findings on multimodal perioperative analgesia, postoperative opioid consumption, and opioid‐sparing strategies [11, 17, 18].

### 4.2. Research Hotspots and Frontiers in PCIA

#### 4.2.1. PCIA‐Related Adverse Reactions and Safety Optimization

As the thematic landscape has evolved, managing adverse reactions and refining safety have become central to PCIA’s transition from a stand‐alone analgesic technique to a pillar of integrated perioperative care. The adverse effects associated with opioid‐based PCIA include PONV, excessive sedation, pruritus, urinary retention, constipation, and gastrointestinal dysfunction. Respiratory depression, although relatively uncommon, demands particular vigilance because of its potentially life‐threatening nature [4, 19–21]. Older patients [19], patients with sleep apnea [22], patients receiving concomitant sedative medications [23], and those undergoing major surgery are at greater risk for excessive sedation, hypoventilation, and altered consciousness. With nonopioid adjuvants increasingly incorporated into PCIA regimens, studies have begun to examine their capacity to mitigate opioid‐related adverse events, while also flagging possible hemodynamic, gastrointestinal, renal, and neuropsychiatric safety concerns. Tailoring the balance between analgesic benefit and safety risk therefore remains essential.

Looking across the hotspot network, it becomes apparent that PCIA safety research distributes its attention unevenly across risk types. Themes such as PONV/nausea, opioid‐related adverse reactions, and opioid‐sparing adjuvant combinations are clearly visible in the current knowledge map. By contrast, respiratory depression, excessive sedation, urinary and gastrointestinal dysfunction, pump programming errors, PCIA by proxy, inadequate monitoring, and process‐related failures receive comparatively little visibility. This skewed coverage suggests that low‐frequency but high‐risk events, as well as systemic process vulnerabilities, deserve more systematic focus in future work. Importantly, the safety of PCIA is not determined solely by the choice of drug. It also hinges on equianalgesic dose conversions, background infusion settings, demand dose and lockout interval, concomitant medications, patient education, and the frequency of nursing surveillance [24]. Clinical implementation, then, must attend to drug selection, compatibility, pump parameter configuration, patient risk stratification, and postoperative monitoring procedures. Moreover, adverse‐effect profiles differ across opioids [25], so simply comparing analgesic intensity or total opioid consumption cannot fully capture the clinical value of a given PCIA regimen [26]. Clinical practice guidelines for postoperative pain management reinforce that patients receiving systemic opioid analgesia should have their sedation level, respiratory status, and other adverse events monitored closely, particularly in the early postoperative window or after dose adjustment [27]. Future PCIA studies would benefit from standardizing the reporting of common adverse effects and systematically capturing low‐frequency but high‐risk safety endpoints—respiratory depression, excessive sedation, device‐related errors, PCIA by proxy, inadequate monitoring, and process‐related risks. Clear definitions of adverse events, specified monitoring time windows, and consistent reporting standards would substantially improve the comparability and clinical interpretability of safety evaluations across different PCIA regimens.

#### 4.2.2. Multimodal and Opioid‐Sparing PCIA Strategies

Multimodal, opioid‐sparing approaches to PCIA have emerged as a defining direction in this evolving landscape. The central aim is to lower opioid exposure and the associated adverse‐event burden without sacrificing analgesic adequacy—by combining agents with complementary mechanisms and integrating other perioperative analgesic measures—ultimately enhancing recovery quality and patient experience [5]. Pharmacologically, the literature has largely concentrated on pairing opioids with nonopioid analgesics such as *α*_2_‐adrenoceptor agonists, NMDA receptor antagonists, and NSAIDs. Dexmedetomidine, in particular, has become a signature adjuvant in PCIA in recent years. Evidence suggests it may improve postoperative analgesia while exerting an opioid‐sparing effect, though vigilance for bradycardia and hypotension remains warranted [28–30]. Esketamine, an NMDA receptor antagonist, offers mechanistic complementarity with opioids and has consequently become a prominent focus of combination‐regimen research in PCIA [31, 32]. Nonopioid analgesics such as NSAIDs and acetaminophen remain key pillars of multimodal analgesia; their use, however, should be tailored to each patient’s bleeding risk, renal and hepatic function, gastrointestinal risk profile, and the specific surgical context [33–35].

Beyond drug combinations confined to the pump, pairing PCIA with regional analgesic techniques has drawn considerable attention. The scope of PCIA research now extends well beyond intravenous agents, increasingly emphasizing the fusion of systemic analgesia with targeted local pain control. Regional blocks deliver site‐specific, procedure‐tailored analgesia, while PCIA supplies continuous, adjustable systemic coverage; together, they offer complementary layers of pain relief [36, 37]. In thoracic and abdominal surgery, cesarean section, and other settings where moderate‐to‐severe postoperative pain is expected, such combined strategies may reduce systemic opioid demand and rescue analgesia use, while improving dynamic pain control and the overall analgesic experience. Outcomes, however, are modulated by the choice of block, local anesthetic regimen, type of surgery, and baseline PCIA formulation [38–41]. Taken together, multimodal, opioid‐sparing PCIA strategies mitigate opioid‐related risks while preserving adequate analgesia by exploiting mechanistic complementarity, synergy with regional techniques, and procedure‐specific design. Future work should test whether optimized PCIA regimens can curtail opioid exposure without undermining analgesic efficacy or safety.

### 4.3. Challenges in Clinical Implementation and Outcome Evaluation of PCIA Research

The real‐world value of PCIA hinges not only on the analgesic regimen chosen but equally on the quality of its implementation and the standardization of outcome assessment. At present, the published literature still underreports key details such as implementation workflows, resource utilization, and patient‐centered endpoints, making it difficult to judge the clinical utility and broader feasibility of different PCIA strategies. First, PCIA is inherently patient‐driven. Its clinical success depends on more than drug selection and pump settings; it is also shaped by preoperative education, the patient’s grasp of device operation and safety constraints, the rigor of nursing surveillance, and overall process management [3, 34, 42]. Prior work has indicated that structured PCIA education may improve patients’ understanding of the device and its use, yet its impact on pain scores, opioid consumption, and recovery trajectories awaits more rigorous confirmation [43–45]. Future studies should therefore report, in a more explicit and reproducible manner, the content, timing, mode of delivery, and adherence to patient education, so that the true contribution of educational interventions to PCIA effectiveness and patient experience can be ascertained. Second, resource consumption associated with PCIA should not be reduced to the costs of pumps, disposables, and drugs alone. Process‐related and outcome‐related expenditures—drug preparation, pump programming, nursing monitoring, acute pain service follow‐up, rescue analgesia, and the management of adverse reactions—must also be accounted for [46–49]. Incorporating direct, process, and outcome‐related resource consumption into evaluations would yield a more complete picture of the clinical value and health‐economic implications of different PCIA regimens. Finally, patient satisfaction and the need for additional or rescue analgesics are essential markers of patient‐centered effectiveness. Postoperative pain management guidelines and core outcome set initiatives in perioperative pain research consistently underscore that analgesic assessment should not rest on pain scores or opioid consumption alone; patient experience, functional recovery, and adverse events carry equal weight [33, 50]. Yet existing PCIA studies still provide sparse detail on patient satisfaction, consumption of additional analgesics, the number of rescue‐analgesic events, and time to first rescue analgesia. Relevant meta‐analyses have likewise documented limited and heterogeneous reporting of these outcomes [29]. Moving forward, investigators should standardize the reporting of patient education, medical costs, rescue analgesia, satisfaction, and functional recovery, so that the clinical merits and optimal application scenarios of different PCIA strategies can be evaluated more comprehensively [50, 51].

### 4.4. Limitations

Several limitations of this study deserve mention. First, the literature search was confined to the WOSCC. While this database offers notable strengths in citation analysis and coverage of high‐impact journals, it does not capture studies indexed solely in Scopus, PubMed, or Embase. The findings should, therefore, be read as a map of PCIA research as represented within WOSCC—primarily reflecting publication trends, collaboration patterns, citation structures, and hotspot shifts visible through that lens—and not as a substitute for systematic reviews, meta‐analyses, or evidence syntheses drawn from multiple databases. Second, restricting inclusion to English‐language publications may have led to an underestimation of contributions from non‐English‐speaking settings to both clinical practice and research on PCIA. Third, the clustering, burst detection, and network visualizations generated by VOSviewer and CiteSpace are inevitably shaped by algorithm parameters, keyword consolidation, and the normalization of author and institution names; some degree of information or interpretation bias cannot be fully excluded. Finally, bibliometric analysis principally captures research attention and the movement of thematic hotspots; it does not equate to the strength of clinical evidence, nor can it replace systematic reviews, meta‐analyses, or cost‐effectiveness studies that target sharply defined clinical questions. Consequently, this study cannot directly establish how a particular PCIA strategy affects patient satisfaction, analgesic requirements, adverse event rates, or healthcare costs. Rigorously designed prospective and multicenter investigations are needed to address these gaps and to further clarify the clinical value and appropriate application contexts of different PCIA regimens. Despite these limitations, the present work provides an overarching view of the global research landscape and hotspot evolution in PCIA, and it may offer a useful reference point for subsequent focused reviews, clinical trial design, and broader perioperative pain management research.

## 5. Conclusion

This study provides a systematic overview of the global knowledge architecture and evolving research hotspots in PCIA from 2014 to 2026. Our findings indicate that PCIA research has maintained broadly sustained output, with China, South Korea, and the United States serving as the primary contributors—although publication volume, academic influence, and centrality in international collaboration did not entirely coincide. Research themes have progressively widened from traditional opioid‐based analgesia and efficacy evaluation toward multimodal analgesia, opioid‐sparing strategies, safety optimization, concurrent use with regional blocks, procedure‐specific analgesia, and patient‐centered outcomes. These findings offer a bibliometric summary of the field’s developmental trajectory and may serve to inform future study design and cross‐regional collaboration.

## Author Contributions

Zheng‐Yuan Duan: conceptualization, data management, and writing–original draft. Shuai‐Yu Zhu: visualization and data curation (cleaning and preprocessing). Qi‐Jun Li: visualization and data curation. Xin‐Ya Cao: methodology and writing–review and editing. Xing‐Cheng Wang: formal analysis, software, and validation. Qiu‐Xia Xiao: supervision, writing–review and editing, and resources. Liu‐Lin Xiong: supervision, writing–review and editing, and project administration.

## Funding

This work was supported by the Clinical Key Specialty Construction “Peak Climbing Plan” Project of the Guizhou Provincial Health Commission (No. GZWJWPF2025021), the Young Elite Scientists Sponsorship Program of the Guizhou Association for Science and Technology (to Liu‐Lin Xiong), Zunyi Medical University 12345 Future Talent Training Program‐Technology Elite (No. ZYSE‐2021‐03), the Science and Technology Cooperation Project of the Zunyi Science and Technology Bureau, China (No. Zunshi Kehe HZ [2025]49), and the Talent Research Startup Fund of the First People’s Hospital of Zunyi (to Liu‐Lin Xiong).

## Disclosure

All authors read and approved the final manuscript.

## Ethics Statement

This study did not involve any human participants, animals, or personal data; therefore, ethics approval and consent to participate were not required. The analysis was conducted using publicly available bibliometric data sourced from the Web of Science Core Collection.

## Consent

The authors have nothing to report.

## Conflicts of Interest

The authors declare no conflicts of interest.

## Data Availability

All data generated or analyzed during this study are included in this article. The raw bibliographic records exported from WOSCC and the processed files used for bibliometric analyses are available from the corresponding author upon reasonable request.
